# Evaluation of genetic diversity, agronomic traits, and anthracnose resistance in the NPGS Sudan Sorghum Core collection

**DOI:** 10.1186/s12864-020-6489-0

**Published:** 2020-01-28

**Authors:** Hugo E. Cuevas, Louis K. Prom

**Affiliations:** 1 USDA-ARS, Tropical Agriculture Research Station, 2200 Pedro Albizu Campos Avenue, Mayaguez, 00680 Puerto Rico; 2 USDA-ARS, Southern Plains Agriculture Research Center, College Station, TX 77845 USA

**Keywords:** Anthracnose, Population structure, Genome-wide association analysis, NPGS sorghum germplasm, Genotyping-by-sequencing

## Abstract

**Background:**

The United States Department of Agriculture (USDA) National Plant Germplasm System (NPGS) sorghum core collection contains 3011 accessions randomly selected from 77 countries. Genomic and phenotypic characterization of this core collection is necessary to encourage and facilitate its utilization in breeding programs and to improve conservation efforts. In this study, we examined the genome sequences of 318 accessions belonging to the NPGS Sudan sorghum core set, and characterized their agronomic traits and anthracnose resistance response.

**Results:**

We identified 183,144 single nucleotide polymorphisms (SNPs) located within or in proximity of 25,124 annotated genes using the genotyping-by-sequencing (GBS) approach. The core collection was genetically highly diverse, with an average pairwise genetic distance of 0.76 among accessions. Population structure and cluster analysis revealed five ancestral populations within the Sudan core set, with moderate to high level of genetic differentiation. In total, 171 accessions (54%) were assigned to one of these populations, which covered 96% of the total genomic variation. Genome scan based on Tajima’s D values revealed two populations under balancing selection. Phenotypic analysis showed differences in agronomic traits among the populations, suggesting that these populations belong to different ecogeographical regions. A total of 55 accessions were resistant to anthracnose; these accessions could represent multiple resistance sources. Genome-wide association study based on fixed and random model Circulating Probability (farmCPU) identified genomic regions associated with plant height, flowering time, panicle length and diameter, and anthracnose resistance response. Integrated analysis of the Sudan core set and sorghum association panel indicated that a large portion of the genetic variation in the Sudan core set might be present in breeding programs but remains unexploited within some clusters of accessions.

**Conclusions:**

The NPGS Sudan core collection comprises genetically and phenotypically diverse germplasm with multiple anthracnose resistance sources. Population genomic analysis could be used to improve screening efforts and identify the most valuable germplasm for breeding programs. The new GBS data set generated in this study represents a novel genomic resource for plant breeders interested in mining the genetic diversity of the NPGS sorghum collection.

## Background

Germplasm collections are an important genetic resource used by plant breeders for the development of new crop varieties that are better adapted to different agricultural systems worldwide. Because germplasm collections are large in size, management of these collections for the maintenance of genetic diversity and identification of desired traits or cultivars is a complex task. In fact, there exists a large gap between the genetic diversity present in the germplasm collection and that used in plant breeding programs [1]. To encourage and facilitate the use of germplasm collections, the concept of core collection was introduced in 1984. A core collection comprises a representative subset of approximately 10% of the entire germplasm collection and is used for in-depth phenotypic and genetic analyses [2]. Multiple approaches have been developed and employed to select a core collection with maximum genetic diversity, based on passport information and/or morphological traits [3–5]. Today, germplasm collections are being genetically characterized, based on single nucleotide polymorphisms (SNPs) using approaches such as genotyping-by-sequencing (GBS) [6] to create a unique genetic profile for each accession, which allows the analysis of genetic diversity in the germplasm collection and genetic relationships among the accessions [7–9]. High reproducibility of these SNPs provides the opportunity to compare multiple data sets from different germplasm collections, select accessions based on genotypic information, and associate genomic regions with important economic traits.

Sorghum [*Sorghum bicolor* (L.) Moench] is a highly diverse cereal crop composed of five botanical races (Bicolor, Durra, Caudatum, Guinea and Kafir) characterized by different inflorescence types because of multiple domestication events [10]. Since sorghum is a C_4_ tropical grass, it is known for its high drought tolerance and different agricultural end uses (food, forage, and biomass) [11]. The National Plant Germplasm System (NPGS) of the United States Department of Agriculture (USDA) preserves the largest worldwide sorghum collection consisting of more than 41,860 accessions from 114 countries. Among these accessions, more than 11,000 were collected from the center of origin of sorghum located in northeast Africa, specifically an area extending from Ethiopia and Eritrea to Sudan [12]. The majority of these accessions were collected more than 70 years ago during multiple expeditions; consequently, their passport information is incomplete. Hence, a core collection of 3011 accessions representing 77 countries was established, based on random selection of accessions within the country of origin [13]. However, most of these accessions are photoperiod sensitive (i.e., flower only under short days); therefore, these accessions cannot be evaluated or used in breeding programs located in temperate regions. The present day sorghum breeding programs utilize only a limited portion of the genetic diversity available in the NPGS sorghum collection, whereas the diversity underlying economically important traits remains trapped within the tropical germplasm.

Multiple association panels have been established to capture the genetic diversity present in the NPGS sorghum collection [8, 14–16]. These germplasm resources have been utilized to study grains and bioenergy related traits through the genome-wide association study (GWAS) [15, 17–21]. Nevertheless, these panels comprise converted sorghum lines (i.e., lines adapted to temperate regions) that represent most of the genetic diversity in breeding programs. Genomic characterization of the NPGS Ethiopia core set and Niger collection revealed that a limited portion of the sorghum genetic diversity is present in the association panels [22, 23]. Thus, the characterization of other NPGS core sets is necessary to create a valuable genomic resource for in-depth phenotypic analysis and to expand the genetic diversity of association panels.

Anthracnose, caused by the fungal pathogen *Colletotrichum sublineolum* in Kabat and Bubák (syn. *Colletotrichum graminicola* [Ces.] G.W. Wilson), is a prevalent disease in warm and humid sorghum cultivation regions. In highly susceptible lines, anthracnose can cause substantial yield losses (up to 50%) of both grain and biomass [24]. Several recent studies have identified loci responsible for broad-spectrum resistance to anthracnose in sorghum accessions on chromosomes 5 and 9 [17, 21, 25–27]; however, widespread use of these resistance sources may reduce their durability. On the other hand, the sorghum association panel (SAP) comprises multiple resistance sources that have not been utilized in sorghum breeding programs. However, the low frequency of resistant alleles (< 0.05) makes their detection by GWAS impossible [17, 21]. The combination of genetic diversity present in the SAP and NPGS Ethiopian core set was effective in the identification of a resistance locus present at a low frequency in the SAP [28]. Thus, identification of new resistance loci in the SAP and/or NPGS germplasm collection is necessary to establish a temporal deployment strategy for increasing the durability of anthracnose resistance sources.

The genomic and phenotypic characterization of the NPGS germplasm is necessary to provide sorghum breeders and geneticists the knowledge and genomic tools necessary to utilize and conserve this germplasm. In the current study, the NPGS Sudan core collection was phenotyped for several agronomic traits at two locations and genotyped via GBS to: 1) evaluate its genetic and phenotypic diversity; 2) determine its population structure and its relationship with the SAP; 3) establish its potential use in the analysis of genetic inheritance of important traits using GWAS; and 4) identify new sources of anthracnose resistance and determine their genetic relationship with resistance sources present in the SAP.

## Results

### Genomic diversity of NPGS Sudan core collection

The GBS analysis of the NPGS Sudan core set resulted in the identification of 183,144 SNPs with a frequency higher than 0.05 and an average of one SNP per 3275 kb. Most of these SNPs (157,673) were located within or in proximity (within 5 kb upstream or downstream sequence) of 25,124 annotated genes (Table 1). A total of 11,892 SNPs were non-synonymous, and 7713 SNPs mapped to the 5′ untranslated regions (5’UTRs), suggesting the existence of multiple gene variants. The rate of heterozygosity was less than 0.11 at more than 90% of the SNPs, with an overall average of 0.05, indicating low genetic variation within accessions.

**Table 1:** Genomic distribution of 183,144 SNPs identified among 318 sorghum accessions in the NPGS Sudan core set

	Chromosome										Total
	1	2	3	4	5	6	7	8	9	10	
Overlapped genes	5260	3973	4275	3550	2234	2825	2164	1886	2574	2739	31,480
SNPs											
5’UTR	1183	878	873	812	711	688	535	755	630	648	7713
3’UTR	710	658	655	609	533	516	402	453	472	486	5494
Missense	1420	1536	1309	1218	1600	1032	803	1056	945	972	11,892
Synonymous	1657	1536	1309	1218	1422	1032	803	1056	1102	972	12,109
Intron	2130	1975	2400	2030	1422	1721	1205	1358	1260	1297	16,797
Upstream_gene (5 kb)	6863	5924	6110	5683	4089	4646	3213	3622	4252	4214	48,616
Downstream_gene (5 kb)	7809	7021	6982	6089	4622	5162	4016	3924	4725	4700	55,051
Intergenic	1657	2413	1746	2639	3378	2409	2276	2867	2205	2431	24,020
Others	238	0	436	0	0	0	134	0	157	486	1451

### Population structure of NPGS Sudan core collection

**Fig. 1:**
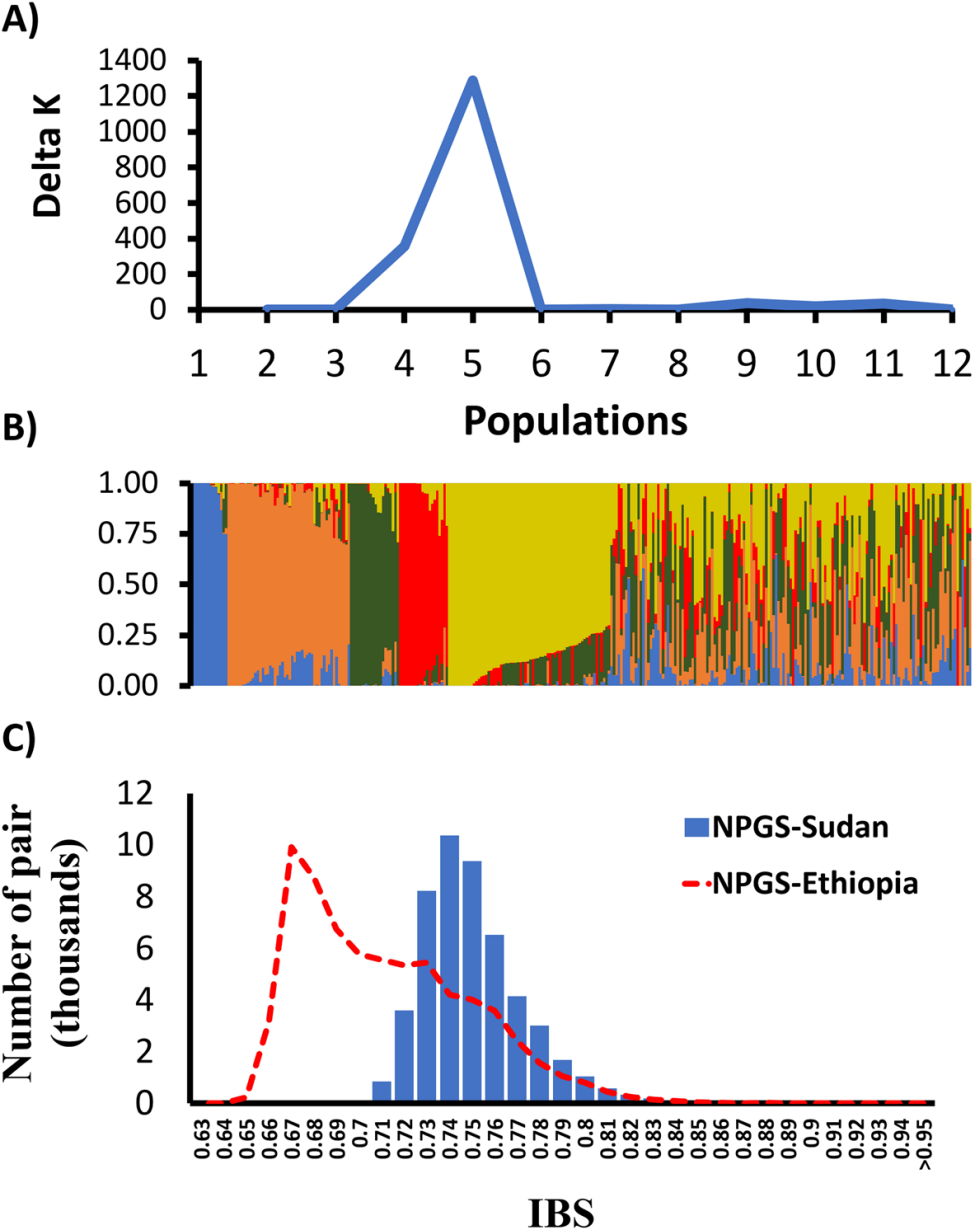
Population structure analysis of the NPGS Sudan core collection. **a** Estimation of number of populations in NPGS Sudan core collection based on the STRUCTURE analysis using 5366 unlinked genome-wide SNP and the Δ*k* values ranging from 1 to 12. The maximum value of Δ*k* suggests the presence of five populations. **b** Hierarchical organization of genetic relatedness of 318 accessions from the NPGS Sudan core set for a K value of five. Each accession is represented by a vertical line partitioned into five colored segments that represent the estimated membership probabilities of the individual to each cluster **c** Distribution of pairwise identity by state (IBS) genetic distance amongst 318 accessions from NPGS Sudan core collection based on the analysis of 5366 unlinked SNPs. Red dashed line represents the distribution of pairwise IBS genetic distance amongst 374 accessions from NPGS Ethiopian collection based on the analysis of 27,306 unlinked SNPs

**Fig. 2:**
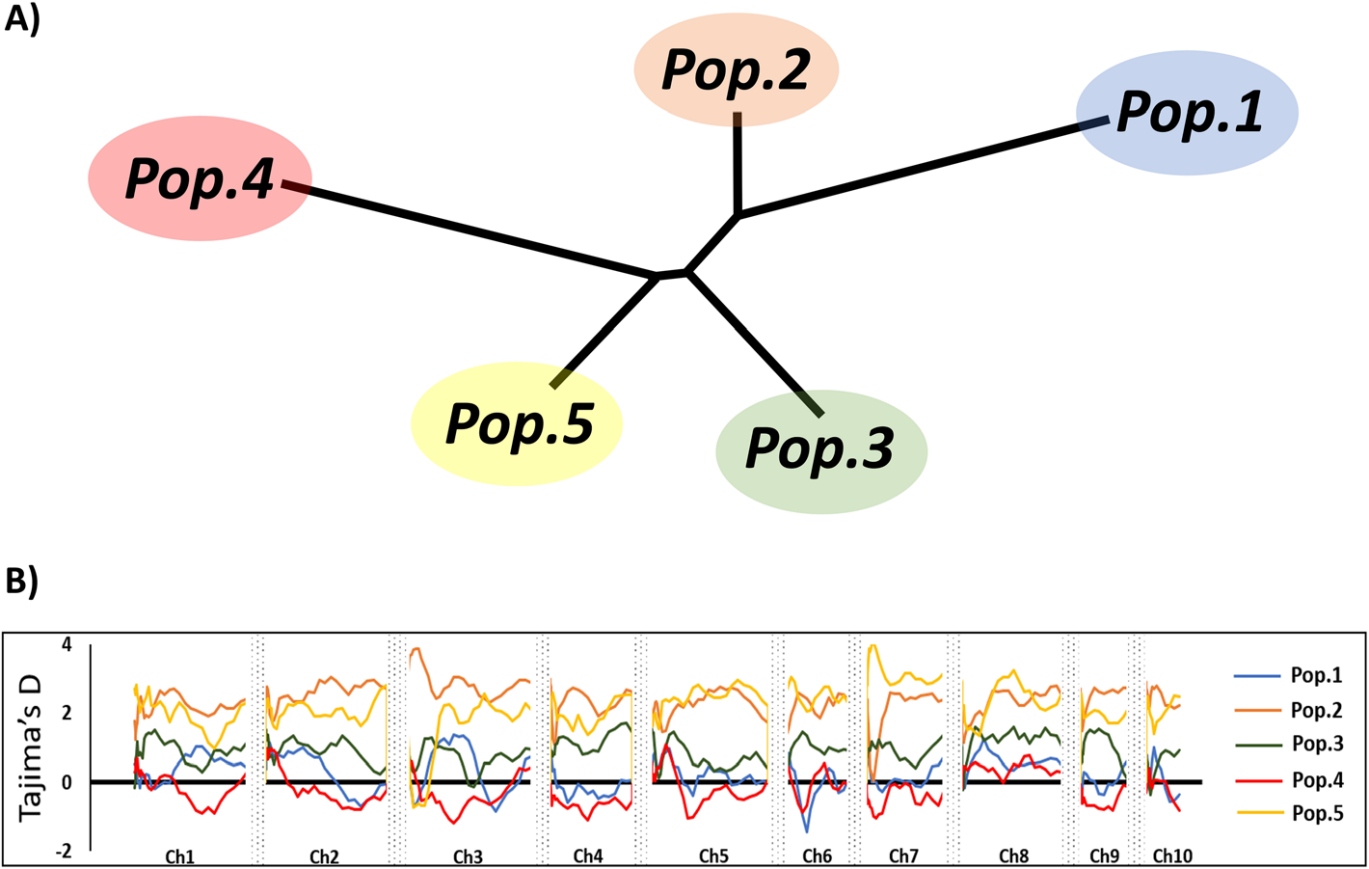
**a** Unrooted neighbor-joining tree based on the F_ST_ genetic differentiation among the five populations present in NPGS Sudan core collection. **b** Genome-wide pattern of Tajima’s D values for 27,244 common SNPs found amongst the five populations present in NPGS Sudan core collection. Each line represents the Tajima’s D values distribution for each population

Population structure analysis of the NPGS Sudan core collection revealed five ancestral populations (Fig. 1a; Additional file 1: Table S2). In total, 171 accessions (54%) were assigned to one of these populations with an ancestry membership coefficient greater than 0.60, while the remaining 147 accessions (46%) showed evidence of admixture (Fig. 1b). The level of genetic differentiation among these five populations ranged from moderate (F_ST_ = 0.17; population 3 vs. population 5) to relatively high (F_ST_ = 0.39; population 1 vs. population 4), indicating that each population must contain accessions with defining traits (Fig. 2a). Indeed, the 171 accessions within the five populations contained 175,388 SNPs (96%), which suggests that the mining of new alleles for sorghum breeding programs should be limited to these accessions.

**Fig. 3:**
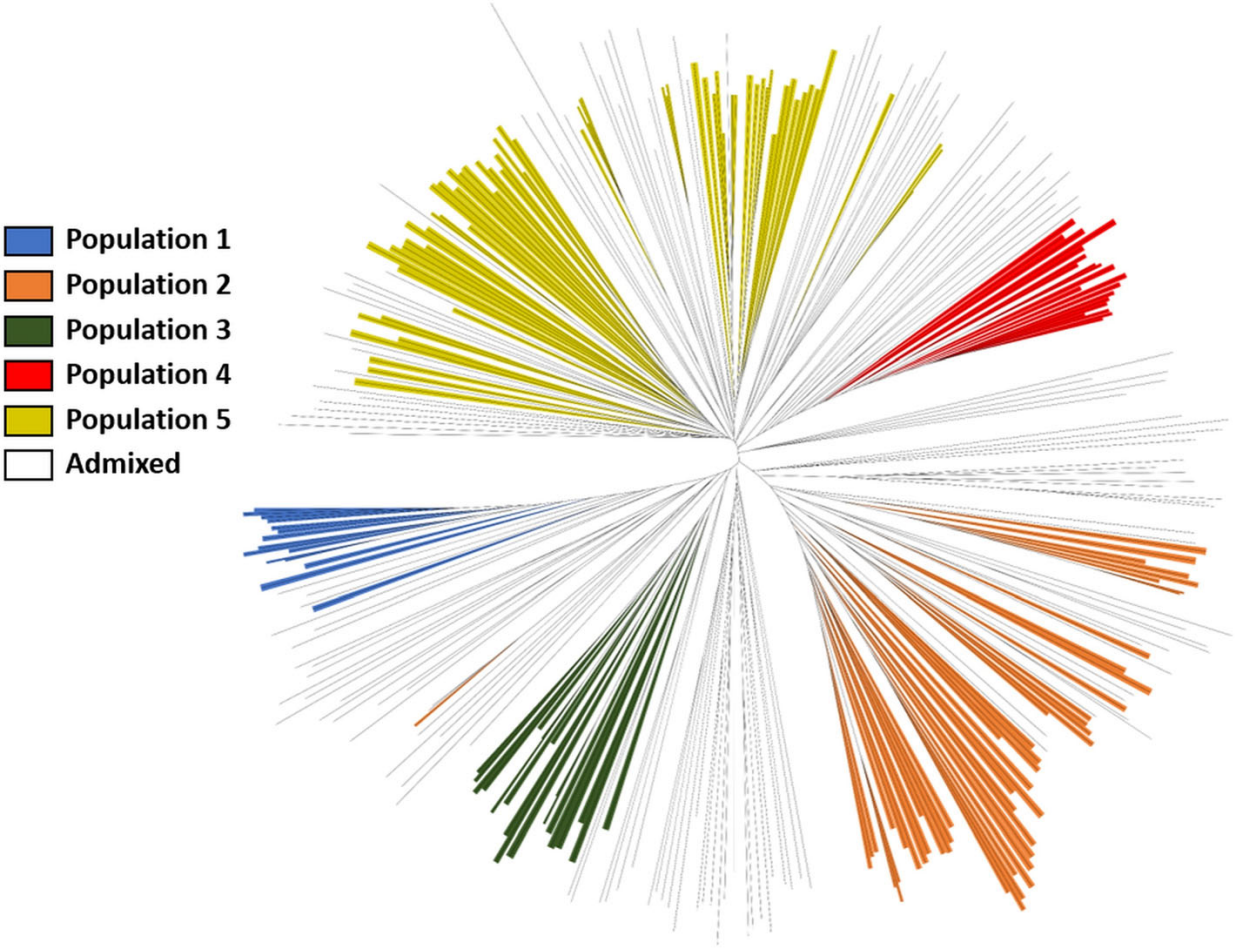
Unrooted neighbor-joining tree of 318 accessions from the NPGS Sudan core collection based on the analysis of 5366 unlinked SNPs. Colored branches represent accessions belonging to the five population present in the NPGS Sudan core collection and admixture accessions are not colored

Pairwise genetic distance among the accessions in the Sudan core set ranged from 0.70 to 0.95 (average = 0.76), indicating that most of these accessions were genetically highly diverse (Fig. 1c). The unrooted neighbor-joining tree supported the previously determined population structure (Fig. 3), with five populations belonging to main clades, which were closely related to admixed accessions. We observed a high frequency of Caudatum genetic background within populations 3, 4, and 5, whereas populations 1 and 2 contained a high frequency of Durra genetic background.

### Allelic diversity in the NPGS Sudan core collection

The Sudan core collection contained 233,404 SNPs, of which 50,260 SNPs were rare (i.e., MAF < 0.05). We found that 97% of these rare alleles were also distributed among the five populations identified by population structure analysis. Moreover, the genetic background of populations was associated with the frequency of rare alleles. The Durra populations 1 and 2 (*n* = 65) contained 43,310 rare alleles, whereas the Caudatum populations 3, 4 and 5 (*n* = 106) contained 27,167 rare alleles.

A total of 4138 private alleles were identified among the populations and admixed groups (Table 2). Consistent with the distribution of rare alleles, the frequency of private alleles was lower in populations 1, 3, 4, and 5 than in population 2. Approximately 86% of all private alleles were present in population 2; thus population 2 was the most genetically diverse population. To explore whether these populations were under differential selection pressure, we determined the Tajima’s D values of genomic regions in these populations. Generally, Tajima’s D value < − 2 indicates positive selection or a selective sweep, whereas Tajima’s D value > 2 is suggestive of balancing selection. Genome scan analysis displayed that populations 2 and 5 were under balancing selection (Fig. 2b). The highest Tajima’s D values were found at the top of chromosome 3 in population 2 and chromosome 7 in population 5. This suggests that these genomic regions are possibly related to adaptive or agronomic traits under positive selection. Increase in the number of accessions in each population is necessary to elucidate the relationship of these private alleles and genomic regions with the evolution of this population structure.

**Table 2:** Private alleles, expected heterozygosity (H_E_), and inbreeding coefficient (F_is_) in the NPGS Sudan core collection

NPGS Sudan core collection	No. of accessions (*n*)	No. of SNPs		H_E_	F_IS_
		Total	Private		
Population 1	15	84,916	109	0.16	0.51
Population 2	50	144,879	3565	0.21	0.73
Population 3	20	11,4375	21	0.19	0.64
Population 4	20	95,768	12	0.15	0.47
Population 5	66	107,692	118	0.20	0.67
Admixed	147	164,430	313	0.25	0.64

**Fig. 4:**
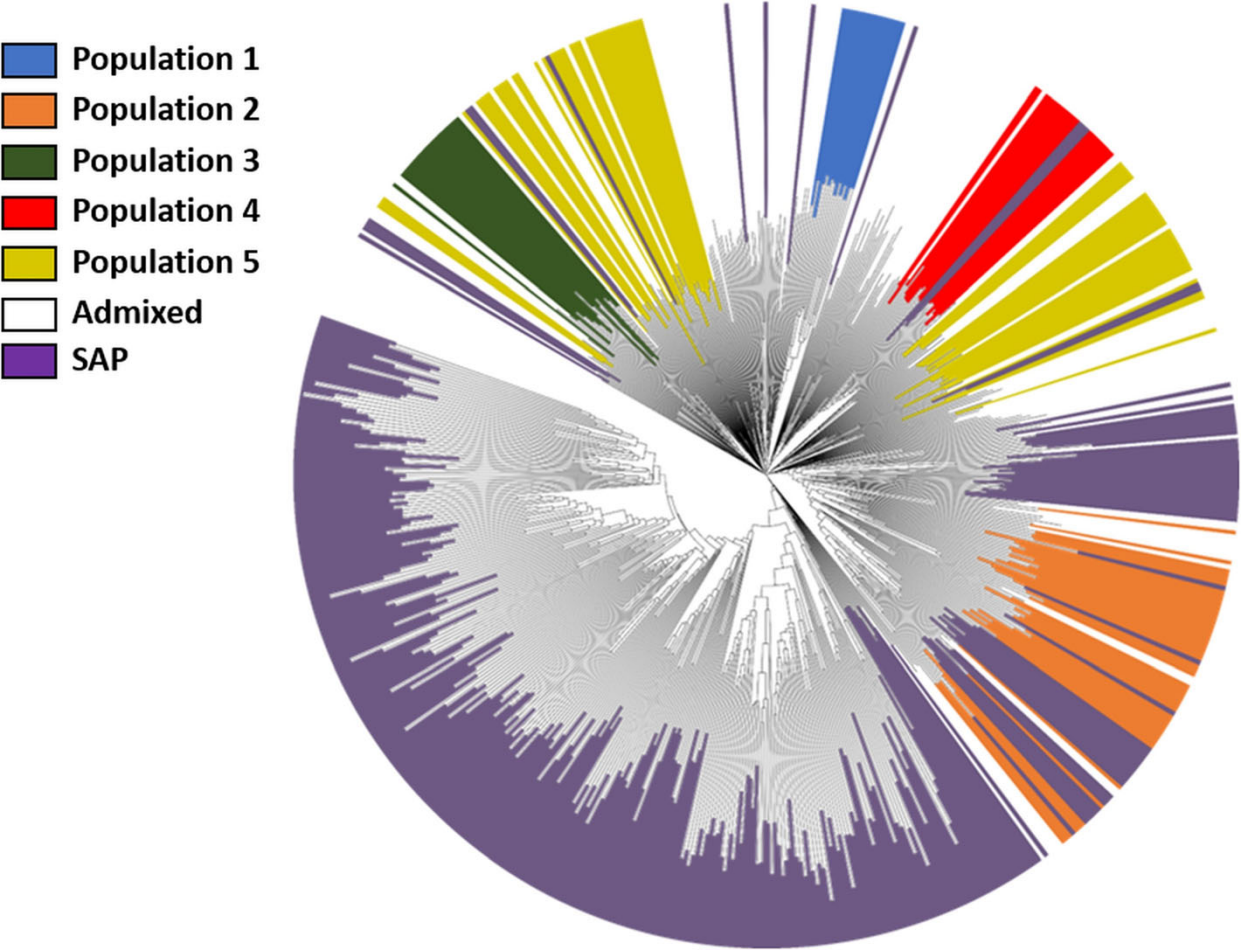
Unrooted neighbor-joining tree of 660 accessions from the NPGS Sudan core collection and sorghum association panel (SAP) based on the analysis of 20,738 unlinked SNPs. Colored branches represent accessions belonging to the five population present in the NPGS Sudan core collection and SAP, while admixture accessions from NPGS Sudan core collection are not colored

To determine the potential use of the Sudan core collection and to expand the genetic diversity in breeding programs, we compared the allelic diversity of the core set with that of the SAP. Most of the private alleles (99.6%) and 28,018 of the rare alleles (56%) in the Sudan core set were identified in the SAP; only thirteen private alleles from population 1 and one private allele from population 5 were absent in SAP. The neighbor-joining cluster analysis of the SAP and Sudan core set, based on 20,738 unlinked SNPs, showed that genetic diversity from population 2 was fully integrated in the SAP (Fig. 4). Twelve accessions from the SAP were observed within populations 4 and 5, while populations 1 and 3 were not represented in the SAP. Our results indicated that a large portion of the genetic variation in the Sudan core set might been used in breeding programs; however, genetic variation within some clusters of accessions remains unexploited.

### Phenotype diversity and anthracnose resistance response in the NPGS Sudan core collection

#### Agronomic traits

The population structure of the Sudan core set was associated with phenotypic variation in six agronomic traits (Table 3; Additional file 1: Table S3). FL, PH, and PL were divided into three groups, while PD and PL/PD ratio were divided into two groups. Variation in PL, PD, and PL/PD ratio was determined by the sorghum race found within each population. Panicle shape was oval in populations 1 and 2, resembling the Durra race, and elongated in populations 3, 4, and 5, as observed in the Caudatum race. However, FL, PH, and midrib color, traits not used to classify sorghum races, differed among populations, reflecting fitness to different environmental conditions within the country. For instance, accessions in population 5 were short and showed early flowering, whereas those in population 2 were tall and showed late flowering. The correlation analysis between FL and PH (*R* = 0.15) indicated that the observed variation in FL and PH is not related to photoperiod sensitivity; thus, differences among populations might be associated with ecogeographical variations within Sudan. Certainly, association of these populations with other important agronomic traits and/or disease resistance would help elucidate the selection process that shaped the population structure and identify valuable accessions for sorghum breeding programs.

**Table 3:** Phenotypic characterization of the five populations in the NPGS Sudan core collection evaluated at Isabela and Mayaguez, Puerto Rico in 2014 and 2016, respectively

Reference accessions	Phenotypic traits^a^					D locus^f^
	Flowering time (days)^b^	Plant height (cm)^c^	Panicle length (cm)^d^	Panicle diameter (cm)^e^	Panicle length/panicle diameter ratio	
RTx430	74.50 ± 2.38	090.83 ± 9.19	20.00 ± 2.50	3.50 ± 0.61	06.05 ± 0.63	*n.a.*
RTx2911	70.40 ± 1.66	093.20 ± 6.37	24.17 ± 1.44	2.33 ± 0.35	10.73 ± 0.56	*n.a.*
BTx623	75.50 ± 1.94	109.00 ± 6.76	18.67 ± 1.61	3.17 ± 0.39	06.13 ± 0.63	*n.a.*
SC748–5	68.13 ± 1.94	091.50 ± 8.72	19.25 ± 1.77	4.75 ± 0.43	04.38 ± 0.69	*n.a.*
SC112–14	61.75 ± 1.94	089.75 ± 7.12	14.50 ± 1.77	4.25 ± 0.43	03.53 ± 0.69	*n.a.*
*Sudan germplasm*						
Population	***	***	***	***	***	*n.a.*
Location	***	***	***	***	***	*n.a.*
Population × Location	**	n.s.	n.s.	n.s.	n.s.	*n.a.*
Population 1	59.40 ± 1.23 bc	151.63 ± 5.25 abc	22.52 ± 1.24 a	4.18 ± 0.26 ab	5.83 ± 0.36 a	1.00
Population 2	65.12 ± 0.67 a	153.02 ± 2.95 ab	19.20 ± 0.61 abc	4.46 ± 0.13 a	4.53 ± 0.18 b	0.49
Population 3	61.47 ± 1.07 b	145.15 ± 4.35 bc	17.99 ± 0.94 bc	3.43 ± 0.20 b	5.77 ± 0.27 a	0.86
Population 4	58.28 ± 0.97 bc	137.72 ± 4.29 c	16.28 ± 0.85 c	3.73 ± 0.18 b	4.60 ± 0.24 ab	0.94
Population 5	56.83 ± 0.55 c	142.79 ± 2.25 c	17.98 ± 0.50 bc	3.95 ± 0.10 b	4.83 ± 0.14 ab	0.36
Admixture	59.65 ± 0.55 b	159.00 ± 1.75 a	19.46 ± 0.37 ab	4.06 ± 0.08 ab	5.17 ± 0.11 a	0.82

^a^Data represent mean ± standard error (SE). Different lowercase letters indicate significant differences (*p* < 0.05; Tukey-Kramer HSD test)

^b^Flowering time refers to the number of days until 50% of the plants in a plot reached anthesis

^c^Plant height refers to the distance from the base of the main stalk to the top of the panicle

^d^Panicle length refers to the distance from the base to the top of the panicle

^e^Panicle diameter refers to the widest region of the panicle

^f^D locus refer to the frequency of colorless midrib

n.s., ** and *** refers to no significant, and significant effects at *p* ≤ 0.01, and 0.001, respectively

#### Anthracnose resistance response

The anthracnose resistance phenotype of the Sudan core accessions indicated that most accessions were susceptible to anthracnose (*X* = 3.44). A total of 55 accessions (22%) with disease incidence scores ≤2.0 across experiments were classified as resistant (Additional file 1: Table S3). Previous studies showed most of the accessions classified as resistant in Puerto Rico were resistant at Georgia and Texas, USA [17, 29]. Thus, further screening at other locations could be limited to these 55 resistant accessions.

To obtain insights into the relationship between Sudan population structure and anthracnose resistance response, we compared the incidence of anthracnose among populations. The disease incidence in population 3 (*X* = 2.76) was lower than that in population 1 (*X* = 4.21) (Table 4), whereas the disease incidence in populations 2, 4, and 5 was similar (*X* = 3.44 to 3.54). Remarkably, disease incidence in populations 2, 4, and 5 was not different from that in populations 1 and 3. We observed evidence of selection for anthracnose resistance in population 3 and existence of resistant accessions (*X* < 2.0) in other populations.

**Table 4:** Anthracnose incidence in the five populations present in the NPGS Sudan core collection evaluated in 2014, 2016, and 2017 at Isabela and Mayaguez, Puerto Rico

NPGS Sudan core set	No. of accessions (n)	Disease incidence^a^
Population 1	13	4.21 ± 0.31 a
Population 2	40	3.44 ± 0.18 ab
Population 3	17	2.76 ± 0.27 b
Population 4	19	3.51 ± 0.26 ab
Population 5	60	3.55 ± 0.15 ab
Admixed	124	3.17 ± 0.10 b

^a^Data represent least square mean ± SE. Different lowercase letters indicate significant differences (*p* < 0.05; Tukey-Kramer HSD test)

#### Genome-wide association study

**Fig. 5:**
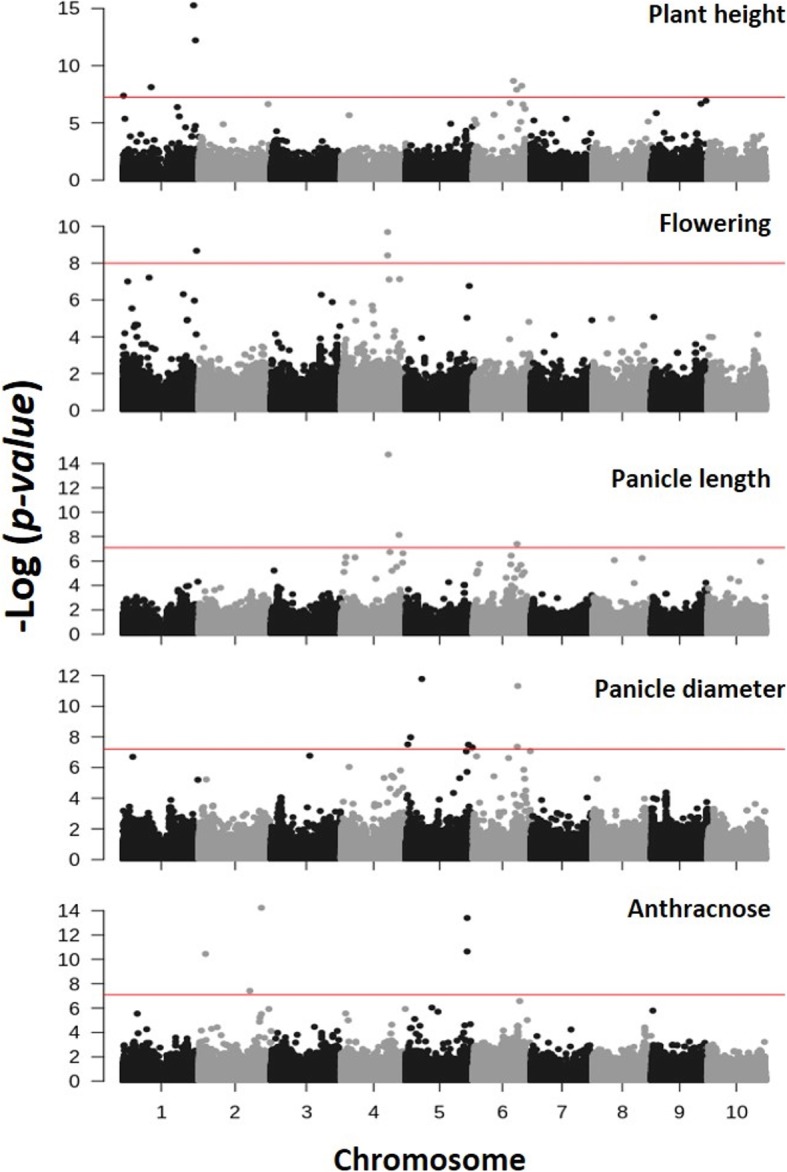
Manhattan plots for the genome-wide association study (GWAS) of plant height, panicle length and diameter, flowering time and anthracnose resistance in the NPGS Sudan core collection based on the analysis of 183,184 SNPs. GWAS based on the fixed and radom model Circulating Probability Unification (farmCPU) regression analysis using the bonferroni-corrected threshold with 0.01 (red line)

Genetic characterization of the NPGS Sudan core collection provides a new genomic resource for the investigation of important agronomic traits. To validate the accuracy of genomic data and its potential use in GWAS, we first analyzed the Dry Stalk (D) locus, which determines the midrib color in sorghum, as shown recently by GWAS and biparental mapping [30]. Logistic regressions found associations between the same previously identified genomic region and the two most significant SNPs [S6_50895868 (*p* = 7.83E-09) and S6_50902627 (*p* = 1.10E-08)], flanking the causal gene Sobic.006G147400 (Additional file 2: Figure S1). The results of Q-Q plots indicated that the first four vectors from the PCA improved the control of genetic relatedness and decreased the chance of spurious associations. The farmCPU analysis for FL, PH, PL, PD and anthracnose resistance found 23 genomic regions associated with these traits (Table 5; Fig. 5). The results of Q-Q plots indicated the inclusion of the ancestry membership coefficient for five population is adequate to decrease the chance of spurious associations (Additional file 3: Fig. S2). The results of GWAS indicated that agronomic traits could be study in the NPGS Sudan core set, but the inclusion of other diversity panels could aid to increase the power to detect other loci.

**Table 5:** Genomic regions associated with four agronomic traits and anthracnose resistance based on the genome-wide association study (GWAS) of the NPGS Sudan core collection. GWAS based on the fixed and radom model Circulating Probability Unification (farmCPU) regression analysis with 183,184 SNPs

Trait	Chr.	LD Block		-Log (*p-value*)	R^2^
		Start	End		
Plant height^a^	1	416,331	434,057	7.37	0.04
	1	29,783,762	29,783,794	8.12	0.01
	1	74,967,854	74,979,878	15.26	0.04
	1	76,839,774	76,874,261	12.21	0.01
	6	42,487,962	42,487,963	8.66	0.04
	6	45,989,817	45,989,940	7.90	0.02
	6	51,222,126		8.23	0.02
Panicle length^b^	4	49,996,017		14.73	0.28
	4	61,404,967	61,418,574	8.15	0.01
	6	46,298,267		7.40	0.11
Panicle diameter^c^	5	2,065,748		7.51	0.01
	5	5,040,622	5,041,030	7.97	0.01
	5	16,909,747	16,933,693	11.78	0.04
	5	66,459,474	66,470,917	7.49	0.05
	5	70,640,897		7.31	0.05
	6	46,520,827	46,535,258	7.36	0.07
	6	47,060,908		11.32	0.02
Flowering^d^	1	78,035,708		8.67	0.08
	4	49,228,258	49,258,333	9.69	0.20
Anthracnose^e^	2	6,699,292	6,699,316	10.45	0.02
	2	53,789,238		7.42	0.02
	2	66,059,468	66,061,773	14.24	0.02
	5	65,136,879	65,137,882	13.40	0.07

^a^Plant height refers to the distance from the base of the main stalk to the top of the panicle

^b^Panicle length refers to the distance from the base to the top of the panicle

^c^Panicle diameter refers to the widest region of the panicle

^e^Flowering time refers to the number of days until 50% of the plants in a plot reached anthesis

^f^Anthracnose resistance response according to Prom et al. 2009

##### GWAS flowering

The GWAS for FL found association with two genomic regions in chromosome 1 and 4. Both genomic regions account for up to 28% of the observed variation. The region in chromosome 4 explains 20% of the observed variation and is constituted by 30 kb block with four genes (Sobic.004G155600 – Sobic.004G155900). These genes are annotated as encoding heat shock proteins, ribonucleoprotein, acetolactate synthase and uncharacterized proteins. The associated SNP in chromosome 1 was located 200 bp downstream of gene (Sobic.001G512633) encoding an uncharacterized protein.

##### GWAS plant height

The GWAS for PH found association with seven genomic regions located in chromosome 1 and 6 (four and three loci, respectively). Each of these genome regions explains 1–4% of the observed variation, however, together account for up to 18%. Two genomic regions in chromosome 6 were 315 kb upstream and 3.19 Mb downstream from the *Dw2* dwarfing gene located in the 42.8 Mb region [31]. Amongst the multiple candidate genes within associated genomic regions, one locus in chromosome 6 constituted by the SNP S6_51222126 is located 2.4 kb upstream of the gene Sobic006G151500 that encode a cytokinin dehydrogenase. A orthologues gene in rice regulated plant height, panicle size and tillering [32].

##### GWAS panicle length

The GWAS for PL found association with two genomic regions in chromosome 4 and one genomic region in chromosome 6. One genomic region in chromosome 4 explains 28% of the observed variation and is constituted by the SNP S4_49996017 located 3.5 kb upstream of the gene Sobic04G157700. This gene encodes the cell wall hydroxyproline-rich glycoprotein. The genomic region in chromosome 6 explains 11% of the observed variation and is constituted by the SNP S6_46298267 located within the gene Sobic06G093000. This gene encodes a methyltransferase.

##### GWAS panicle diameter

The GWAS for PD found association with seven genomic regions located in chromosomes 5 and 6. Each of these genome regions explains 1–7% of the observed variation, and together account for up to 25%. The 14.31 kb genomic region in chromosome 6 explains 7% of the variation and enclose three genes (Sobic06G095100 – Sobic06G095300). Functional annotation of these genes includes OBPS-3-responsive proteins, microsomal signal peptidase and uncharacterized proteins.

##### GWAS anthracnose

The GWAS for anthracnose resistance found association with four genomic regions located in chromosome 2 and 5. Each genomic region in chromosome 2 explains 2% of the observed variation. The SNP S2_6699292 is located within the gene Sobic02G067600 that encode an RNA recognition motif. The other two genomic regions in chromosome 2, SNPs S2_53789238 and S2_66061610, are in two intergenic regions of 189 and 6 kb, respectively. The genomic region in chromosome 5 (SNP S5_651137569) explains 7% of the observed variation and is in a 6 kb intergenic region.

## Discussion

The NPGS sorghum collection is an important public germplasm resource for sorghum breeders and the scientific community worldwide. Nevertheless, screening of this large collection for economically important traits is challenging, expensive, and requires high-throughput phenotyping techniques. Moreover, the photoperiod sensitivity of most of the germplasm limits the screening to tropical regions, where plants can flower under short days. Today, most of the genetic diversity of NPGS collection remains untapped, while most of the improved varieties are genetically related because of inbreeding, and are therefore vulnerable to abiotic and biotic stresses. Understand the phenotypic and genetic diversity that exists among and within germplasm collection enables development of adequate strategies for breeding and allele mining [33].

### NPGS Sudan core collection and breeding programs

Core collections are developed as germplasm resources for the screening and identification of valuable accessions [34]. Nevertheless, most of the accession in the NPGS sorghum core collection lack phenotypic and genotypic information. To access the genetic diversity of the Sudan core set, we genetically characterized these accessions with the GBS platform previously used to genetically characterize the SAP [8] and the NPGS Ethiopian collection [22]. Both the genomic characterization and population structure analysis of Sudan core set provided critical information that was absent during its development but necessary to exploit its genetic diversity in breeding programs. In fact, the collection site was unknown for 66% of the accessions, while the other 24% of the accessions were obtained from the Gezira Research Station, making it impossible to organize the core set based on geographic regions. Therefore, the five populations identified in the Sudan core set provide valuable information for the selection of a reduced number of accessions for further screening. Both Sudan and Ethiopia are considered the center of diversity of sorghum and the area of domestication of races Caudatum and Durra, respectively [11]. Therefore, each population might be associated with the adaptation of both sorghum races to different environmental conditions in Sudan. Several Sudanese accessions have been adapted to temperate regions through the sorghum conversion program [35] and the reinstated sorghum conversion program [36, 37], but neither program has included accessions from the Sudan core set. In this regard, phylogenetic analysis of the Sudan core set with the SAP, allows the selection of the most genetically diverse accessions for adaptation to temperate regions to expand the genetic diversity employed in breeding programs.

### Preservation of NPGS Sudan core collection

Preserve most of the allelic diversity present in a germplasm collection is necessary for the security of agriculture [38]. The success of a core collection is determined by the efficiency of allelic representation and lack of redundancy [39]. Redundancy of accessions is one of the biggest problems in germplasm collections because it requires the maintenance and screening of accessions that do not contribute to the diversity of the collection. Pairwise genetic distance among accessions in the Sudan core collection showed a rather low redundancy, with 39 accessions sharing high genetic similarity (IBS > 0.90). Previous studies showed that core collections based on either genotypic profiles or random selection could represent the genetic diversity of the entire collection [3]. The number of rare alleles (MAF < 0.05) in the Sudan core set (22%) was lower than that observed in the NPGS Ethiopia core set (60% [22]; and NPGS Senegal collection (32% [40];. Thus, the high frequency of the Durra genetic background and high frequency of rare alleles in Sudanese populations 1 and 2 suggests that both of these populations originated from Ethiopia. The number of private alleles depend on the sample size and sampling strategies; therefore, it is difficult to understand the population structure without an adequate sample size [41]. Despite the differences in the number of private alleles among populations, it is possible that populations 1, 3, 4, and 5 were derived from population 2. Although rare alleles are usually not considered for the restoration of endangered species, they are very important for plant breeding programs [42]. The fixation of advantageous alleles reduces polymorphisms in the linked genomic regions, which in turn increases the abundance of rare alleles [43]. The seed increase process in ex situ germplasm collection eliminates potential out-crossing, thus increasing inbreeding and decreasing genetic variability within accessions. We observed a slight reduction in heterozygosity in the Sudan core set compared to the out-crossing rate of sorghum among landraces [44]. The frequency of rare alleles and heterozygosity rate are indicators of genetic diversity that could be used to measure the integrity of the collection during regeneration. At this time, our analysis suggests that most of the genetic variation present in the NPGS Sudan collection should be available in its core set. If more accessions from the NPGS sorghum core collection are genotyped on the same platform, it will be possible to establish a mini core collection [45].

### Genome-wide association study in NPGS Sudan core collection

Genomic characterization of the Sudan core collection could be used to study economically important traits and develop marker–trait association via GWAS. Determination of the population structure is important for the identification of marker-trait association [46]. Population structure of the Sudan core set explained up to 25% of the observed phenotypic variation in quantitative traits including FL, PH, PL, and PD. Since these traits could be associated with environmental niches, the limited number of significant associations in the GWAS could be related to the population structure corrections. The complexity and low heritability of these traits require the use of a larger number of accessions and multiple locations to identify other genomic regions. Likewise, GBS technique cannot cover the whole genome and regions with high recombination rate require larger number of SNPs to be evaluate adequate in the GWAS. Further integration of the Sudan core set with other sorghum diversity panels [8, 16] and NPGS core sets [22] may increase the phenotypic diversity and statistical power for the complete elucidation of these complex traits. Genome scans based on nucleotide polymorphism pattern, environmental variables, selection signature, and candidate gene approach may provide insights into the genetic control of these traits in the Sudanese germplasm [47]. For instance, variation in the photoperiod sensitivity and panicle compactness of the NPGS Senegal collection was associated with several known sorghum genes [40]. Allele frequency distribution of the SNP S4_ 62,316,207 located in the *Tan1* gene (*Sobic.004 g280800* [48]; indicated that genetic variation was limited to the Sudanese populations 1 and 2. An increase in the number of cloned genes in sorghum might enable the selection of a subset of accessions for phenotypic analysis based on haplotype variation.

Multiple algorithms have been developed for GWAS, however, the statistical power of these algorithms disappear for complex traits associated with population structure. FarmCPU method was developed to increase detection power with less false positive than previous algorithm [49]. In fact, GWAS analysis based on the enriched compressed mixed linear model (ECMLM) could not detect association with either evaluated traits. In contrast, farmCPU detected multiple loci for PH, FL, PL, PD and anthracnose resistance suggesting the association of these traits with population structure reduce the statistical power of ECMLM. Although most of the identify loci explain a small portion of the observed variation, they might can have significant effects in breeding populations where population structure is absent. Our study implicated that identify loci for plant height and flowering might be involved in metabolic pathways not associated to photoperiod sensitivity. In fact, either loci identified in the GWAS were associated to previous characterize sorghum maturity and dwarfing genes. The introgression of these loci into temperate adapted germplasm is necessary to determine their potential use in sorghum breeding programs.

In Sudan, sorghum is mostly cultivated in drought-prone areas [50], where environmental conditions are not favorable for fungal diseases. Therefore, anthracnose resistance alleles in the Sudan core populations could be associated with balancing selection, where they have been maintained at a low frequency to protect against anthracnose disease during sporadic wet seasons. Moreover, the divergency among populations (F_ST_ > 0.17) suggests that each population may contain different anthracnose resistance sources. The GWAS identified four loci that might be related to Sudan population structure. We observed allelic variation for the SNP S2_6699313 is limited to populations 2 and 5. Likewise, the genetic variation for the SNP S2_53789238 is limited to population 5, while genetic variation for SNP S2_66061610 is observed in populations 1, 2, 3 and 4. The genetic variation of the SNP in chromosome 5 (S5_65137569) is limited to population 3 and 5. These genomic regions explain a limited portion of the observed variation indicating the presence of other loci. Indeed, 11 resistance accessions could not be associated with these four resistance loci. The statistical power of GWAS for the detection of rare alleles (MAF < 0.05) is very low and decreases further when multiple rare alleles underlying similar phenotypes are present in the selected panel [51]. Most previous inheritance studies of the anthracnose resistance response showed single gene segregation [52–55]. This suggests that the Sudan core set, like the SAP [56], contains multiple low frequency resistance sources that cannot be detected by GWAS.

The fifty-five Sudanese anthracnose resistance accessions identify herein could be used increase the genetic diversity of breeding programs. Phylogenetic analysis showed these resistance accessions represent the genetic variation in the Sudan core collection (Additional file 4: Fig. S3). We observed that twelve resistant accessions in the SAP are genetically related to resistant accessions in populations 2 and 5, indicating that some Sudanese sources of anthracnose resistance are integrated in the SAP. We found that allele frequency of associated SNPs in the SAP and when it was merged with NPGS Sudan core set ranged from 0.08 to 0.45. The absent of association in previous GWAS analysis using SAP could be related to correction of population structure, algorithm (ECMLM vs. fastCPU) or the minor effect of these SNPs over the observe variation. Most of the Sudan and SAP germplasm belong to the Caudatum race, therefore, their combined analysis could not increase the frequency of resistant alleles. For instance, a GWAS that combined 335 NPGS Ethiopian accessions, most of which belonged to the Durra race, and the SAP enabled the detection of a locus on chromosome 9 [28]. Evaluation of the NPGS germplasm belonging to Kaffir and Guinea races, and its further combination with the SAP and NPGS Sudan and Ethiopian core set, should enable the detection of additional resistance loci. Recognition of the origin, evolution, and dispersal of some anthracnose resistance genes will help us understand the variation observed in the resistant response within and among the NPGS core sets.

Sequences in intergenic regions contain enhancer elements that could regulate the expression of genes. These enhancers can be located upstream or downstream of genes, within or far away from genes [57]. We found that three genomic regions associated with anthracnose resistance are in intergenic regions. The prediction of functional elements in intergenic regions is difficult and relied on comparative genomic analysis between species [58]. Further confirmation of these genomic regions based on bi-parental populations could provide insight of its actual role in the anthracnose resistance response. The genomic region in chromosome 5 have been previously associated with anthracnose resistance response in sorghum line SC414-12E [27]. This temperate adapted line derived from a tropical Sudanese accession (PI152621) demonstrating that the use of both bi-parental and GWAS mapping approaches are the most adequate strategy to genomic dissect anthracnose resistance response.

## Conclusion

The results of this study suggest that the NPGS Sudan core collection comprises genetically and phenotypically diverse germplasm with multiple anthracnose resistance sources. Genomic characterization of the core collection revealed that it comprises five populations, whose effective use could expand the genetic diversity of sorghum breeding programs. The SNP data generated in this study provide an objective criterion for the selection of accessions, which could be used for in-depth phenotypic analysis, GWAS, genetic diversity preservation through regeneration, and genetic relationship analysis with other germplasm collections.

## Methods

### Germplasm

A total of 318 accessions in the NPGS Sudan core collection [13] were genetically and phenotypically characterized in this study. This core set represents ~ 13.8% of the total NPGS Sudan collection and consists of accessions belonging to all five known sorghum races, Bicolor (14 accessions), Caudatum (169 accessions), Durra (26 accessions), Guinea (eight accessions), and Kafir (five accessions), as well as intermediate races (96 accessions), according to the Germplasm Resource Information Network (GRIN; Additional file 1: Table S1).

### Field experiment

The core set and five reference breeding lines (RTx430, RTx2911, BTx623, SC748–5, and SC112) were planted at the research farms of the USDA-Agricultural Research Service (ARS) Tropical (18°28′18.6″N 67°02′37.1″W) and Mayaguez (18°12′41.5″N 67°08′08.0″W), Puerto Rico, during the short day-length season (i.e., from September to April) in 2014 and 2016, respectively. A completely randomized design was used at both locations, with plots measuring 1.8 m in length and with 0.9 m spacing between rows. Plants were maintained using standard management practices. Weeds were controlled by mechanical tillage and hand hoeing, and aerial watering (Raingun Irrigation) was applied in the absence of rainfall.

### Phenotypic analysis

Flowering time (FL), plant height (PH), panicle length (PL), panicle diamter (PD), PL/PD ratio, and midrib color were recorded for each plot at both locations. FL was defined as the number of days until 50% of the plants within a plot reached anthesis. PH referred to the average distance from the base of the main stalk at the soil level to the top of the panicle of three plants per plot at maturity. PL and PD referred to the distance from the first rachis to the top of the panicle and the widest section of the panicle, respectively [59]. Data from both locations were combined to perform the analysis of variance (ANOVA) using the *Proc mixed covtest* procedure of SAS (SAS Institute, Cary, NC), where location and accessions were considered fixed effects. Performances of accessions were estimated based on least square means. Midrib color was classified as white, green, and green-yellow at maturity.

### Anthracnose resistance response

High relative humidity (> 80%) at both locations was conducive for the development of anthracnose disease and the evaluation of disease resistance. Five previously characterized pathotypes from Isabela [60] and three pathotypes from Mayaguez were used to prepare two fungal cultures, as described previously (Prom et al., 2009). At both locations, both fungal cultures were used to inoculate plants at 30–40 days after planting. Disease incidence was assessed before harvesting on a scale from 1 to 5 as follows: 1 = no symptoms or chlorotic flecks on leaves; 2 = hypersensitive reaction on inoculated leaves, but no acervuli in the center; 3 = infected bottom leaves with acervuli; 4 = necrotic lesions with acervuli on bottom leaves showing spreading to middle leaves; and 5 = most leaves dead because of infection, and flag leaf infected.

Sixty-six accessions resistant to anthracnose at both locations were evaluated in triplicate for one additional year (2017) together with reference checks at Isabela, Puerto Rico, in a randomized complete block design consisting of three blocks of 1.8 m long plots with 0.9 m spacing between rows. Data of the three experiments were combined to perform ANOVA using the *Proc mixed covtest* procedure of SAS. The location and accession were considered fixed effects, and blocks were treated as random effects. Anthracnose resistance response was estimated based on least square mean.

### GBS

The Sudan core collection was subjected to GBS [6] using the *Ape*KI restriction enzyme, and the data were deposited at the National Center for Biotechnology Information (NCBI) Sequence Read Archive (SRA) database. To determine the genetic variation among accessions, genomic DNA was isolated from a pool of five seedlings, as described previously (P Guillemaut and L Marechal-Drouard [61], and re-purified using the ZR96 DNA Clean & Concentrator-5 (Zymo Research, Irvine, CA, USA). The GBS library was prepared and sequenced in four lanes of an Illumina HiSeq 2500 platform at the Biotechnology Center of the University of Wisconsin, Madison, WI. Sequences were aligned to the BTx623 reference sorghum genome sequence v.3 (www.phytozome.net; accessed February 15, 2018) using bowtie2 [62], while the TASSEL5GBSv2 pipeline [63] was used for SNP calling. Raw sequence reads with minor allele frequency (MAF) > 0.01 and missing data < 0.25 were filtered, thus retaining 260,691 SNPs. Imputations were performed using Beagle 4.1 with probability > 0.80. The imputed genotype data set was filtered for MAF > 0.05 and missing data < 0.80, retaining 183,184 SNPs for GWAS, population structure and phylogenetic analyses. The number of SNPs within genes, in proximity of genes (i.e., within 5 kb upstream and downstream sequences), and in intergenic regions as well as the number of synonymous and no-synonymous SNPs were determined using the Variant Effect Predictor [64], as implemented in Ensembl Plants (plants.ensembl.org; accessed April 2019).

### GBS of accessions in the NPGS Sudan Core set and SAP

Genomic information of 342 accessions from the SAP was obtained from the NCBI SRA database (accession numbers SRX1085248, SRX1085243, SRX1058235, SRX1058234, SRX1058233, SRX1085230, and SRX1085228). Sequences of all 660 accessions (342 accessions in the SAP and 318 accessions in the Sudan core set) were aligned against the BTx623 sorghum genome v.3 using bowtie2 [62], and SNP calling was performed using the TASSEL5GBSv2 pipeline [63]. Raw sequence reads with MAF > 0.01 and missing data < 0.25 were filtered, thus retaining 305,250 SNPs. Imputations were performed using Beagle 4.1 with probability call > 0.80. The imputed genotype data set was filtered for MAF > 0.05 and missing data < 0.80, retaining 209,419 SNPs.

### Population structure analysis of the NPGS Sudan core set

A pruned subset of 5366 unlinked SNPs (*r*^*2*^ < 0.10) was generated using PLINK [65] and used to determine population structure and admixed ancestry using the model-based clustering method implemented in STUCTURE 2.1 [66]. Three independent runs using an admixture model with correlated frequencies, 50,000 burn-in periods, and 125,000 Monte Carlo Markov Chain (MCMC) for each *k* value set from 1 to 13 were performed using STRUCTUE. The ad hoc statistic Δ*k* based on the rate of change in the log probability of data between successive *k* values [67], as implemented by the Structure Harvester software [68], was used to deduce the number of populations in the core set. These three runs were matched by permutation in CLUMPP [69], and accessions with an ancestry membership coefficient < 0.60 were considered admixed.

### Genetic diversity and cluster analysis

The number of private SNPs was estimated among accessions in the Sudan core collection and SAP [8] and among the Sudan core set populations. The expected heterozygosity [70], inbreeding coefficient, and pairwise fixation index (F_ST_) [71] among the Sudan core set populations were estimated based on 5366 unlinked SNPs in the R package Hierfstat [72]. Tajima’s D values were estimated based on a sliding window, with a bin and step size of 500 and 100 kb, respectively, and 27,244 common SNPs, as implemented in TASSEL 5.2 [73]. Pairwise genetic distance among the 318 Sudanese accessions was calculated based on identity-by-state (IBS), as implemented in TASSEL 5.2 [73]. The resulting matrix was subject to clustering analysis using the neighbor-joining method and visualized using the Interactive Tree of Life [74].

### GWAS

The GWAS was conducted based on the phenotypic data of FL, PH, PL, PD, PL/PD ratio, and anthracnose resistance with the fixed and random model Circulating Probability (farmCPU) model using the GAPPIT package [75] in the R software. In the farmCPU, the ancestry membership coefficient from STRUCTURE were included to control for population structure and family relatedness. Plant midrib color was analyzed as a binary trait (i.e., white vs. green and green-yellow). A multivariate logistic regression model was fitted to the binary data using PLINK [65], with the first four components of a principal components analysis (PCA) and population structure included as covariates to control for family relatedness.

The PLINK [65] *block* function was used to delimited genomic regions related to associated SNPs. Log quantile-quantile (QQ) *p-*value plots were examined to determine how well farmCPU and logistic regression accounted for population structure and family relatedness. Manhattan and QQ plots were visualized using the R package qqman [76].

### Significance threshold

Empirical significance thresholds for farmCPU were based on Bonferroni with experiment-wise error rate of *p* = 0.01 as implemented in GAPPIT. The empirical significance threshold for logistic regressions with the midrib data using the Sudan core set (−log_10_ [*p*-value] = 5.42) was calculated with 1000 permutations for an experiment-wise error rate of *p* = 0.05.

## Supplementary information

**Additional file 1: Table S1.** List of sorghum accessions from the USDA-NPGS Sudanese core collection. **Table S2.** Hierarchical organization of the genetic relatedness of 318 accessions from the USDA-NPGS Sudan germplasm collection based on the population structure analysis of 5000 unlinked SNPs. **Table S3.** Anthracnose resistance response and agronomic traits of USDA-NPGS Sudan sorghum core collection

**Additional file 2: Figure. S1.** Genome-wide association study (GWAS) of midrib color in the NPGS Sudan core collection. **(A)** Manhattan plot for logistic regression based on case-control analaysis (i.e., white midrib vs. others), and significance threshold of *p-value*s < 0.05 and < 0.001 based on 1000 permutations. **(B)** Log quantile-quantile (Q-Q) *p*-value plots

**Additional file 3: Figure. S2.** Log quantile-quantile (Q-Q) *p*-value plots for the genome-wide association study (GWAS) of plant height, panicle length and diameter, flowering time and anthracnose resistance in the NPGS Sudan core collection. GWAS based on the fixed and radom model Circulating Probability Unification (farmCPU) regression analysis

**Additional file 4: Figure. S3.** Unrooted neighbor-joining tree of 55 anthracnose resistance accessions present in NPGS Sudan core collection based on the analysis of 5366 unlinked SNPs. Colored branches represent accessions belonging to the five population present in the core collection and admixture accessions are not colored

## Data Availability

The datasets generated for this study can be found in the National Center for Biotechnology Information (NCBI) Sequence Read Archive (https://www.ncbi.nlm.nih.gov/sra) under the BioProject PRJNA565973. The germplasm used in this study can be obtained from USDA-NPGS germplasm collection through the Germplasm Resource Information Network (https://www.ars-grin.gov).

## Abbreviations

ANOVA: Analysis of variance
ECMLM: Enriched Compressed Mixed Linear Model
farmCPU: fixed and random model Circulating Probability
FL: flowering time
*F*_ST_: fixation index
GAPIT: Genome Association and Prediction Integrated Tool
GBS: Genotyping-by-sequence
GWAS: Genome-wide association studies
H_E_: Expected heterozigosity
IBS: Identity by state
MAF: Minor allele frequency
NPGS: National Plant Germplasm System
PCA: Principal component analysis
PD: Panicle diameter
PH: Plant height
PL: Panicle length
PL/PD: Ratio length/diameter of panicle
Q–Q: Quantile–quantile
SAP: Sorghum association panel
SNP: Single-nucleotide polymorphism
USDA: United States Department of Agriculture
