# Supplementary figures and images for: Evaluation of genetic diversity, agronomic traits, and anthracnose resistance in the NPGS Sudan Sorghum Core collection
Source: BMC Genomics. 2020 Jan 28;21:88. doi: 10.1186/s12864-020-6489-0 (PMC6988227; doi:10.1186/s12864-020-6489-0)

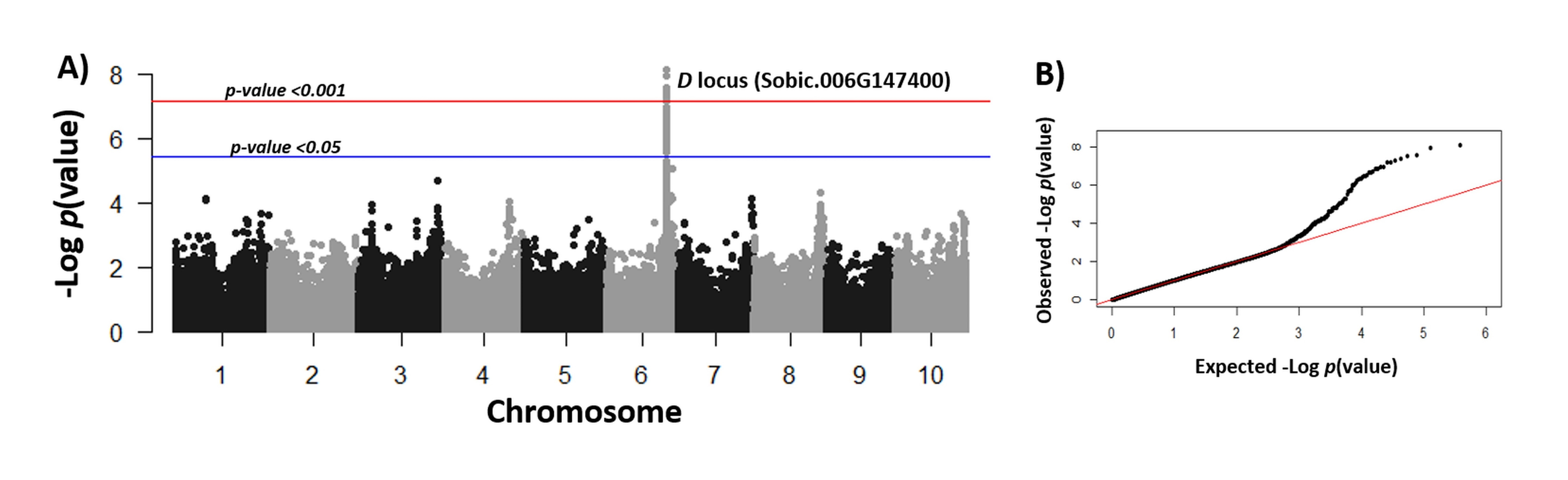

Supplement: Supplementary file 2 — Additional file 2: Figure. S1. Genome-wide association study (GWAS) of midrib color in the NPGS Sudan core collection. (A) Manhattan plot for logistic regression based on case-control analaysis (i.e., white midrib vs. others), and significance threshold of p-values < 0.05 and < 0.001 based on 1000 permutations. (B) Log quantile-quantile (Q-Q) p-value plots [file 12864_2020_6489_MOESM2_ESM.jpg]

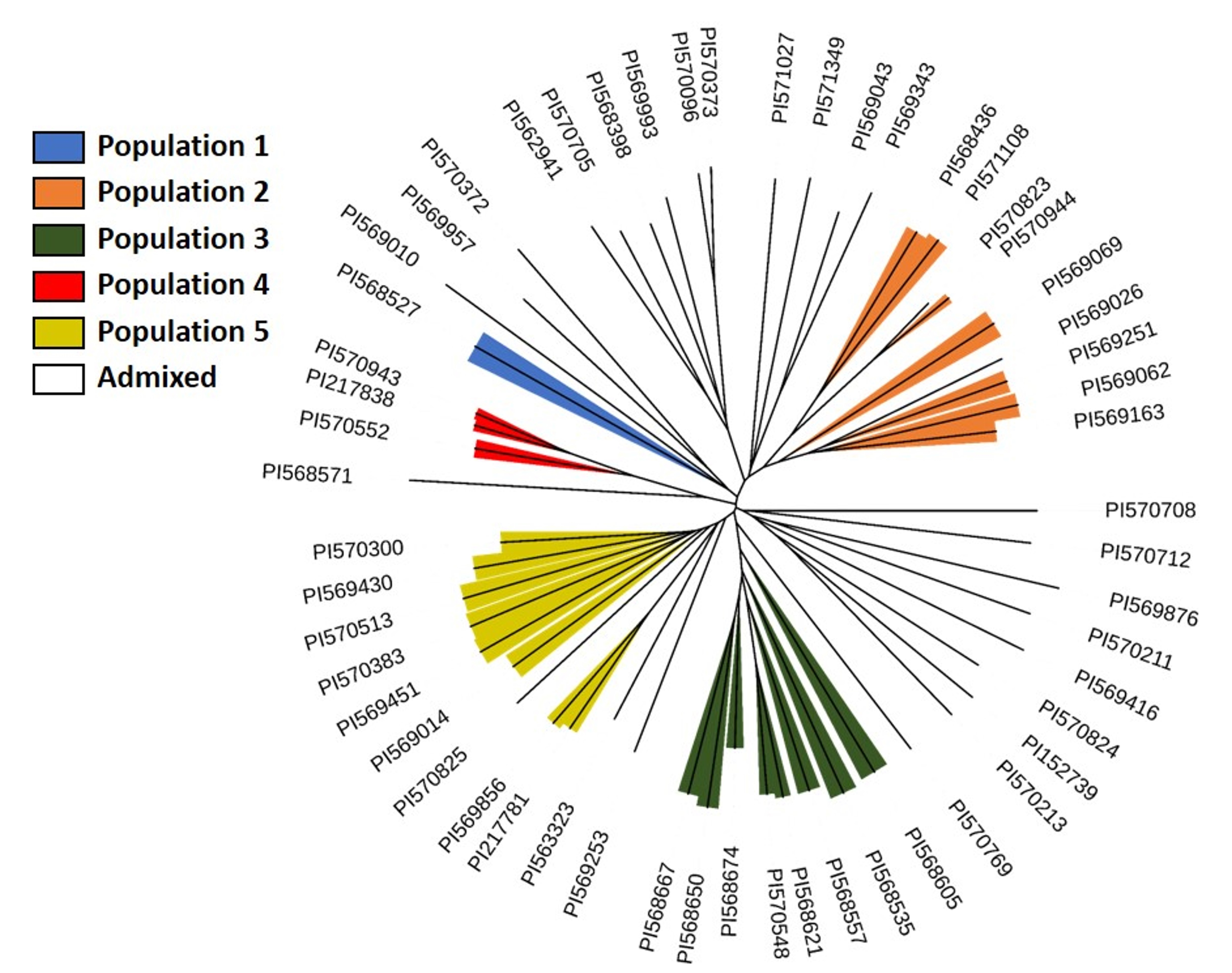

Supplement: Supplementary file 4 — Additional file 4: Figure. S3. Unrooted neighbor-joining tree of 55 anthracnose resistance accessions present in NPGS Sudan core collection based on the analysis of 5366 unlinked SNPs. Colored branches represent accessions belonging to the five population present in the core collection and admixture accessions are not colored [file 12864_2020_6489_MOESM4_ESM.jpg]
